# Unveiling Gender Characteristics in Pakistan: Forensic Dentistry Insights through Panoramic Radiographs and Morphometrics Analysis

**DOI:** 10.1055/s-0045-1806945

**Published:** 2025-04-23

**Authors:** Huma Sarwar, Urooba Mirza, Sarah Mariam Anwar, Meshal Muhammad Naeem, Juzer Shabbir, Tazeen Zehra, Azam Shahzad, Muhammad Sohail Zafar

**Affiliations:** 1 Department of Operative Dentistry, Dr. Ishrat-ul-E bad Khan Institute of Oral Health, Karachi, Pakistan; 2 Department of Operative Dentistry, Dr. Ishrat-ul-E bad Khan Institute of Oral Health Sciences, Karachi, Pakistan; 3 Department of Periodontology, Dr. Ishrat-ul-E bad Khan Institute of Oral Health Sciences, Karachi, Pakistan; 4 Department of Operative Dentistry, Baqai Dental College, Baqai Medical University, Karachi, Pakistan; 5 Department of Operative Dentistry, Liaquat College of Medicine and Dentistry, Karachi, Pakistan; 6 Department of Dental Materials, Shahida Islam Dental College, Lodhran, Punjab; 7 Department of Clinical Sciences, College of Dentistry, Ajman University, Ajman, United Arab Emirates; 8 Centre of Medical and Bio-allied Health Sciences Research, Ajman University, Ajman, United Arab Emirates; 9 School of Dentistry, University of Jordan, Amman, Jordan

**Keywords:** radiographs, forensic, gender, identification, mandible

## Abstract

**Objectives:**

To validate the use of panoramic radiographs and morphometric parameters in forensic odontology for accurate and efficient gender determination in the specific socio-cultural context of the Pakistani population.

**Materials and Methods:**

A retrospective study was conducted using orthopantomograms from 130 individuals aged between 16 and 30 years, sourced from a radiology department. The study included comprehensive measurements of mandibular indices such as condylar height, coronoid height, and antegonial depth. Measurements were made using Image J software. The indices were analyzed through univariate, multivariate, and best models to assess their effectiveness in gender prediction. Statistical analysis included independent samples *t* -test, binary logistic regression, and receiver operator characteristic (ROC) analysis to evaluate threshold values, sensitivity, specificity, and area under the curve (AUC) for each index.

**Statistical Analysis:**

Independent samples *t* -test was used to compare the means of indices with gender. Binary logistic regression was used to estimate the likelihood of male gender, and ROC analysis was used to calculate threshold values, sensitivity, specificity, and AUC.

**Results:**

Univariate analysis revealed that most indices, except for the gonial angle, showed significant differences between genders. The multivariate model stated the condylar height and coronoid height as a significant predictor. The best model confirmed condylar height, coronoid height, antegonial depth, and the inferior border of the mental foramen as reliable indices for male gender determination. The ROC demonstrated that the distance from the mean inferior border to the lower border of the mandible had the highest AUC of 82%, indicating strong predictive power.

**Conclusion:**

The study confirmed the effectiveness of specific mandibular measurements in gender determination within the Pakistani population. Condylar height, coronoid height, antegonial depth, and the inferior border of the metal foramen are consistently significant predictors across various models. Further research with a larger population sample is recommended.

## Introduction

Within the field of forensic odontology, one of the most valuable and intriguing tasks is determining the age and gender of a deceased person. In modern times, availability of large-scale and extremely efficient databases has remarkably facilitated human identification in postmortem circumstances. Forensic odontology has become a prime instrument at crime scenes, explosions, warfare, aviation disasters, and in the identification of missing persons. 1 Skeletal structures such as skull, pelvis, and cranium bones are important tools for identifying human remains with considerable accuracy. 2

The use of radiographic imaging provides a reliable method for dental record keeping. In mass disaster situations, forensic odontologist plays a very important role in victim identification, especially when high-quality ante-mortal dental records are available. 3 In Pakistan, dental practitioners exhibit inadmissible practices regarding the maintenance and keeping of records and histories related to dental patients, whether it be computer-based or manual. Despite understanding the value of dental record keeping, there is a general laxity among these practitioners toward maintaining records. 4 When ante-mortal records are unavailable, mandibular radiographs may be an important tool in sex determination by utilizing the sexual dimorphism inherent in mandibular bone. 5 The morphological differences in the bone pattern in males and females help forensic odontologists distinguish between both genders and provide supportive information for human identification. 5

The most significant geometric morphometric differences between males and females are found in the pelvis, followed by the humerus and cranium. 6 In the cranium, the mandible is the largest and most robust facial bone. The morphology of the mandible is impressively preserved in forensic and anthropological studies. The anatomy of the mandible plays a crucial role in differentiating between ethnic populations and determining sexual dimorphism. The lower jaw is an extremely solid and highly resilient bone that lends itself brilliantly to forensic sciences and anthropology. This bone is built to resist high mechanical stress and is, therefore, able to withstand physical trauma including fractures and burns. These physical features guarantee that the lower jaw will very often remain intact and recognizable even after severe conditions. This exceptionally high durability of the mandible makes it a valid means of classifying human remains. 7 Several mandibular parameters have been proposed for determining gender from forensic remains. These include metrics like bicondylar width, inter-coronoid separation, and other vertical or horizontal dimensions that are visible on panoramic radiographs. 8 Additionally, studies utilizing dental anatomy parameters, such as stature estimation and crown diameters, provide a broader context for forensic and anthropological assessments. 9

Machine learning offers significant potential in mandibular morphometric analysis by automating feature extraction and enhancing the accuracy of sex determination. If sex determination is required, a population-specific morphometric standard is necessary, and advanced algorithms can identify subtle skeletal variations, enabling forensic experts to develop precise classification models. 10 Therefore, if a judgement is to be made for differentiating between the sexes, the criteria must be distinct and developed specifically for population in question, rather than relying on generalized population data for the measurements. 11 Gender determination is a crucial aspect of forensic science, aiding in the identification of human remains, especially in cases of mass disasters or unidentified bodies. Skeletal features, particularly the mandible and cranium, exhibit distinct characteristics that help forensic experts differentiate between sexes with accuracy. Advancements in forensic imaging and morphometric analysis have improved sex determination methods, emphasizing the need for population-specific standards. In regions with limited dental record-keeping, establishing reliable skeletal classification criteria enhances the accuracy of forensic investigations. This study focuses on addressing the gaps in the literature by providing population-specific threshold values for gender determination using mandibular parameters in Pakistan. Therefore, the aim of this study was to evaluate the accuracy and validity of mandibular morphometric parameters in gender determination.

## Materials and Methods

A retrospective study was conducted using orthopantomograms (OPGs) of 130 individuals, sourced from the Radiology Department at Dr. Ishrat ul Ebad Khan Institute of Oral Health Sciences, Karachi, Pakistan. The sample size was calculated using the online sample size calculator for sensitivity and specificity studies available at https://wnarifin.github.io/ssc/sssnsp.html . Based on an assumed sensitivity of 88.97% and specificity of 81.38% from a prior study, the required minimum sample size for this study was determined to be 130. This calculation ensured adequate statistical power for evaluating the diagnostic accuracy of mandibular morphometric analysis in sex determination. OPGs of participants aged between 16 and 30 years, exclusively from Pakistani decedents, were selected. The data were stratified by gender.

The inclusion criteria for this study required panoramic radiographs captured between January 2023 and May 2023, with available patient gender and demographic details. Only fully dentate patients with no missing teeth in the mandibular region were included to ensure accurate morphometric analysis. Additionally, optimal quality OPGs with clear visualization of anatomical landmarks were selected to maintain measurement precision. Conversely, radiographs were excluded if exhibited poor quality, including motion artifacts, distortion, or low resolution that affected landmark visibility. Cases with incomplete visualization of the mental foramen or other critical anatomical structures were also excluded. Furthermore, radiographs showing bilateral asymmetry of the mandible or the presence of fractures, pathology, or deformities that could alter morphometric parameters were excluded. This rigorous selection process ensured that only diagnostically reliable images were used, enhancing the accuracy of sex determination in mandibular morphometric analysis.

All mandibular measurements were conducted using ImageJ software, an open-source image processing tool widely used for biomedical image analysis. ImageJ enables precise measurement of anatomical structures by allowing users to calibrate images, apply segmentation techniques, and extract dimensional data from radiographs. Its accuracy and reproducibility make it a reliable tool for analyzing mandibular metrics in forensic and anthropological studies.

**Fig. 1:**
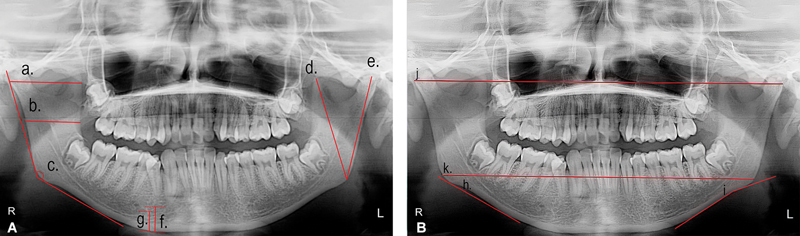
A representative orthopantomogram showing various anatomical landmarks: ( **A** ) a. Maximum ramus width; b. minimum ramus width; C. Gonial angle; d. coronoid height; e. Condylar height; f. distance from superior border of mental foramen to inferior border of mandible; g. distance from inferior border of mental foramen to inferior border of mandible. ( **B** ) h. Antegonial depth; i. antegonial angle; j. bicondylar width; k. bigonial width.

Some parameters were measured bilaterally in millimeters ( Fig. 1A ). To assess the maximum ramus width, which is the greatest anteroposterior diameter of the mandibular ramus measured from the most anterior point to a line connecting the most posterior point on the condyle with the angle of the jaw, the image was opened in image J and the “straight line” tool from toolbar was selected. 12 13 14 15 16 17 18 A cursor was placed at the starting, clicked and dragged to the endpoint representing the maximum ramus width. The measurement displayed in the results window was then recorded. Similarly, the minimum ramus width is defined as the smallest anteroposterior width of the mandibular ramus that is determined by locating the distance between its anterior and posterior aspects along the horizontal axis of the ramus. 12 13 14 15 16 17 18 The “straight line” tool was used to measure this distance, following the same procedure described for measuring the maximum width. Condylar height and coronoid height were measured by identifying the uppermost points of the condyle and the coronoid process respectively and the lowest points on the inferior body of the ramus. 13 14 15 16 17 18 19 20 21 22 The “straight line” was used to measure the vertical distance for both parameters. The antegonial angle was assessed using the “Angle” tool by positioning the cursor at the anterior part of the mandible's inferior border and the deepest point of the gonion. 19 The resulting angle displayed in the results window was recorded. For the gonial angle, the “Angle” tool was used to construct two lines, one tangent to the ramus and mandibular condyle, and one tangent to the most inferior point of the gonial region and the body of the mandible. 14 15 18 19 21 22 The angle seen in the results window was noted. Antegonial depth is measured as the vertical distance from the lowest part of the lower edge of the mandible to its deepest concavity. 19 23 This depth was measured as the perpendicular distance from the deepest point of the antegonial notch to a line drawn parallel to the lower cortical border of the mandible using the “straight line” tool. Some parameters were also measured in millimeters using Image J software but not bilaterally ( Fig. 1B ). To assess the distance from the superior and inferior borders of the mental foramen to the inferior border of the ramus, the “straight line” tool was selected and the linear distance between the specific points was recorded. 24 Lastly, the bigonial and bicondylar widths were assessed using the “straight line” tool. 12 15 22 For the bigonial width, the horizontal distance between the two gonials, from right gonion to left gonion, was measured. For the bicondylar width, the distance was calculated between the rearmost points of the posterior border of the right and left condyle. The resulting measurements displayed in the results window were recorded for both parameters. The intra- and inter-rater reliability was assessed with the help of the intra-class correlation coefficient (ICC). The ICC value of at least 0.8 was considered satisfactory.

### Statistical Analysis

**Fig. 2:**
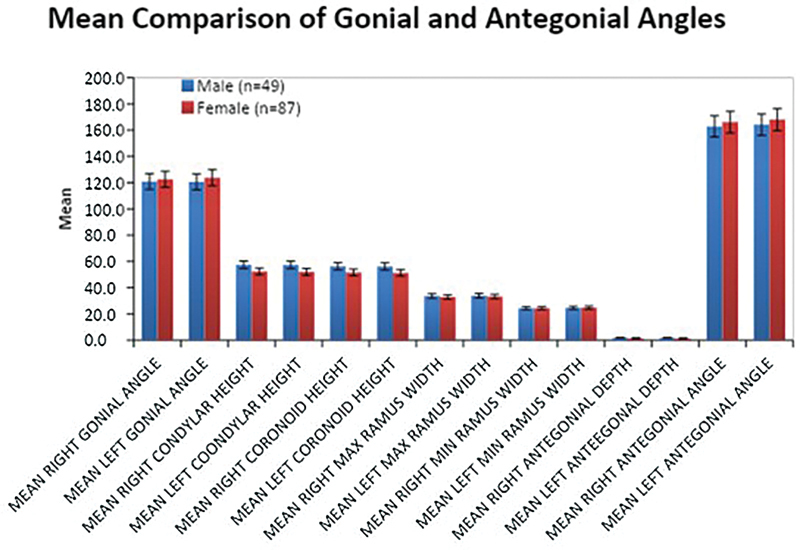
Mean comparison of gonial and antegonial angles.

Data were stored and analyzed using IBM-SPSS version 23.0; counts with percentages were reported for male and females; means with standard deviation were given for age (years), right, left, and total (mean of both sides) gonial, antegonial, and other measured indices. The data were normally distributed (as shown by normality tests such as the Shapiro–Wilk test with a *p* -value >0.05). Independent samples *t* -test was used to compare the means of these indices with gender ( Fig. 2 ). Binary logistic regression analysis was used to estimate the likelihood of the male gender by both univariate and multivariate models adjusted with age and other studied indices. The forward LR method based on the maximum likelihood of the index was finally used to obtain the best model for predicting gender. Odds ratios with 95% confidence intervals were reported for all indices. Receiver operator characteristic (ROC) analysis was conducted to find out the threshold values of indices, sensitivity, specificity, and area under the curve (AUC) with its significance for the probability of determining correctly male gender was also reported. All these analyses were performed for the right, left, and both sides of the measured indices. *p* -Values less than 0.05 were considered statistically significant.

## Results

The sample consisted of 136 individuals, of which 64% ( *n* = 87) were females and 36% ( *n* = 49) were males. The mean age of the participants was 22.4 years (standard deviation = ± 2.4), ranging from 16 to 26 years. The inter- and intra-rater reliability was found to be >0.8

Table 1 presents the mean values of gonial and antegonial angles along with condylar height, coronoid height, and other related indices for both genders. The comparison of mean values indicates that males had a significantly lower left gonial angle (120.6 ± 8.2°) compared with females (123.7 ± 6.8°; *p* = 0.018). However, the right gonial angle did not differ significantly between genders ( *p* = 0.23). The condylar height was found to be significantly higher in males, with a mean right condylar height of 57.4 ± 5.6 mm compared with 52.4 ± 3.5 mm in females ( *p* < 0.0), and a left condylar height of 57.3 ± 6.1 mm in males compared with 52.0 ± 4.0 mm in females ( *p* < 0.01). A similar trend was observed for coronoid height, with significantly higher mean values in males on both the right (56.2 ± 5.3 mm) and left (56.1 ± 5.5 mm) sides compared with females (51.6 ± 3.6 and 51.3 ± 3.9 mm, respectively; *p* < 0.01).

**Table 1:** Mean comparison of gonial and antegonial angles with gender

Parameters	Male ( *N* = 49)		Female ( *N* = 87)		*p* -Value a
	Mean	SD	Mean	SD	
Mean right gonial angle	120.9	8.4	122.5	7.3	0.23
Mean left gonial angle	120.6	8.2	123.7	6.8	0.018 b
Mean right condylar height	57.4	5.6	52.4	3.5	<0.01 b
Mean left condylar height	57.3	6.1	52.0	4.0	<0.01 b
Mean right coronoid height	56.2	5.3	51.6	3.6	<0.01 b
Mean left coronoid height	56.1	5.5	51.3	3.9	<0.01 b
Mean right max ramus width	33.6	3.7	32.9	3.6	0.31
Mean left max ramus width	33.8	3.6	33.3	3.0	0.34
Mean right min ramus width	24.4	2.6	24.4	2.8	0.93
Mean left min ramus width	24.7	3.2	24.8	2.7	0.79
Mean right antegonial depth	1.9	1.0	1.5	0.9	0.007 b
Mean left antegonial depth	1.9	1.1	1.4	0.8	0.002 b
Mean right antegonial angle	162.9	10.2	166.1	7.9	0.047 b
Mean left antegonial angle	164.2	9.6	168.1	6.9	0.015 b

Abbreviation: SD, standard deviation.

Note: Coronoid height, left coronoid height, right antegonial depth, and left antegonial depth of male samples were significantly higher than those of female samples ( *p* < 0.05), whereas mean for right antegonial angle and left antegonial angle of male samples was found significantly lower than females ( *p* < 0.05); there was no significant mean difference obtained for right gonial angle, right max ramus width, left max ramus width, right min ramus width, and left min ramus width between male and females ( *p* > 0.05).

a Analyzed with the help of independent sample *t* -test.

b Statistically significant.

Additionally, antegonial depth was significantly greater in males, with a mean right antegonial depth of 1.9 ± 1.0 mm compared with 1.5 ± 0.9 mm in females ( *p* = 0.007), and a left antegonial depth of 1.9 ± 1.1 mm in males compared with 1.4 ± 0.8 mm in females ( *p* = 0.002). The antegonial angles followed an inverse trend, with males having significantly lower right (162.9 ± 10.2°) and left (164.2 ± 9.6°) antegonial angles compared with females (166.1 ± 7.9° and 168.1 ± 6.9°, respectively; *p* < 0.05). No significant differences were found in maximum ramus width, minimum ramus width, or right gonial angle between the two groups ( *p* > 0.05).

Table 2 further confirms these findings for total (mean of left and right) values. Males had a significantly higher superior border of mental foramen (14.9 ± 1.8 mm) than females (12.8 ± 1.6 mm; *p* < 0.05), and the inferior border of mental foramen was also significantly higher in males (11.9 ± 1.6 mm) than females (9.8 ± 1.5 mm; *p* < 0.05). Bigonial width was significantly greater in males (157.0 ± 10.4 mm) than in females (150.0 ± 9.2 mm; *p* < 0.05), whereas bicondylar width was significantly lower in males (173.0 ± 11.8 mm) than in females (177.9 ± 8.4 mm; *p* = 0.006). Condylar height and coronoid height were also significantly higher in males ( *p* < 0.05). The antegonial depth remained higher in males (1.9 ± 1.0 mm) than in females (1.4 ± 0.8 mm; *p* = 0.002), while the antegonial angle was lower in males (163.5 ± 9.0°) than in females (167.1 ± 6.8°; *p* = 0.01). Gonial angle, maximum ramus width, and minimum ramus width showed no statistically significant differences ( *p* > 0.05).

**Table 2:** Mean comparison of total gonial and antegonial angles with gender parameters

	Male ( *N* = 49)		Female ( *N* = 87)		*p* -Value a
	Mean	SD	Mean	SD	
Mean superior border of mental foramen	14.9	1.8	12.8	1.6	<0.01 b
Mean inferior border of mental foramen	11.9	1.6	9.8	1.5	<0.01 b
Mean bicondylar width	173.0	11.8	177.9	8.4	0.006 b
Mean bigonial width	157.0	10.4	150.0	9.2	<0.01 b
Gonial angle	120.7	8.0	123.1	6.8	0.07
Condylar height	57.3	5.7	52.2	3.6	<0.01 b
Coronoid height	56.2	5.2	51.5	3.6	<0.01 b
Max ramus width	33.7	3.2	33.1	2.9	0.26
Min ramus width	24.5	2.5	24.6	2.5	0.84
Antegonial depth	1.9	1.0	1.4	0.8	0.002 b
Antegonial angle	163.5	9.0	167.1	6.8	0.01 b

Abbreviation: SD, standard deviation.

a Analyzed with the help of independent sample *t* -test.

b Statistically significant.

Table 3 summarizes the results of binary logistic regression models for predicting male gender based on individual indices. The univariate model indicated a significant positive association between male gender and increasing condylar height, coronoid height, and antegonial depth for both the right and left sides ( *p* < 0.05). Additionally, in the total indices model, the mean superior border of the mental foramen, mean inferior border of the mental foramen, and mean bigonial width were significantly associated with the male gender ( *p* < 0.05). The univariate model also showed that the left antegonial angle, mean bicondylar width, total gonial angle, and total antegonial angle had a significant negative association with the male gender ( *p* < 0.05).

**Table 3:** Estimation of models to predict male gender

Side	Indices	Univariate model OR (95% CI)	Multivariate model OR (95% CI)	Best model forward LR stepwise
Right	Gonial angle	0.97 (0.92–1.01)	1.06 (0.98–1.14)	
	Condylar height	1.27 a (1.15–1.39)	1.17 a (1.01–1.36)	1.27 a (1.15–1.39)
	Coronoid height	1.28 a (1.15–1.42)	1.20 a (1.01–1.44)	
	Max ramus width	1.05 (0.95–1.16)	0.98 (0.79–1.21)	
	Min ramus width	0.99 (0.87–1.13)	0.88 (0.69–1.13)	
	Antegonial depth	1.70 a (1.13–2.56)	1.88 (0.77–4.55)	
	Antegonial angle	0.96 (0.92–1.00)	1.02 (0.93–1.12)	
Left	Gonial angle	0.94 (0.89–0.99)	0.99 (0.92–1.06)	
	Condylar height	1.23 a (1.13–1.34)	1.13 (0.99–1.30)	1.25 a (1.14–1.38)
	Coronoid height	1.25 a (1.14–1.38)	1.18 a (1.00–1.39)	
	Max ramus width	1.05 (0.94–1.17)	1.07 (0.88–1.30)	
	Min ramus width	0.98 (0.87–1.11)	0.79 (0.63–1.00)	
	Antegonial depth	1.83 a (1.20–2.77)	1.27 (0.48–3.36)	
	Antegonial angle	0.94 a (0.89–0.98)	0.95 (0.85–1.06)	0.91 a (0.86–0.97)
Total (mean of right and left)	Mean superior border of mental foramen	2.02 a (1.54–2.64)	0.82 (0.45–1.47)	
	Mean interior border of mental foramen	2.41 a (1.75–3.32)	3.19 a (1.50–6.76)	2.66 a (1.68–4.20)
	Mean bicondylar width	0.99 a (0.99–1.00)	0.95 (0.90–1.01)	0.99 a (0.99–1.00)
	Mean bigonial width	1.07 a (1.03–1.12)	1.11 a (1.04–1.18)	1.12 a (1.03–1.21)
	Gonial angle	0.95 a (0.90–1.00)	1.03 (0.95–1.11)	
	Condylar height	1.26 a (1.15–1.38)	1.16 a (1.00–1.35)	1.20 a (1.04–1.38)
	Coronoid height	1.29 a (1.16–1.44)	1.22 a (1.01–1.47)	
	Max ramus width	1.07 (0.95–1.20)	1.03 (0.83–1.29)	
	Min ramus width	0.98 (0.85–1.13)	0.79 (0.61–1.03)	0.73 a (0.55–0.96)
	Antegonial depth	1.90 a (1.21–2.97)	1.71 (0.54–5.41)	2.62 a (1.33–5.15)
	Antegonial angle	0.94 a (0.89–0.98)	0.99 (0.87–1.12)	

Abbreviations: CI, confidence interval; OR, odds ratio.

a OR considered statistically significant with *p* < 0.05.

Multivariate analysis identified significant predictors of male gender. On the right side, condylar height and coronoid height were significant ( *p* < 0.05), while on the left side, only coronoid height remained significant. For total indices, the mean inferior border of the mental foramen, mean bigonial width, condylar height, and coronoid height were significant predictors of male gender ( *p* < 0.05). The best model, using the forward stepwise logistic regression method, identified right condylar height, left condylar height, left antegonial angle, mean inferior border of the mental foramen, mean bicondylar width, mean bigonial width, total condylar height, total minimum ramus width, and total antegonial depth as significant predictors of male gender ( *p* < 0.05).

Table 4 presents the results of ROC analysis to determine threshold values, sensitivity, specificity, and AUC for various indices in predicting male gender. Among the right-side indices, coronoid height had the highest accuracy, with an AUC of 77.0%, a sensitivity of 70.8%, and a specificity of 68.6% ( *p* < 0.05). The corresponding threshold value for coronoid height was 53.0 mm. On the left side, coronoid height also demonstrated a high predictive value, with an AUC of 76.1%, a sensitivity of 66.7%, and a specificity of 65.1% ( *p* < 0.05), with a threshold of 52.8 mm. The best overall predictor was the mean inferior border of the mental foramen, which had an AUC of 82.9%, a sensitivity of 77.1%, and a specificity of 76.5% ( *p* < 0.01), with a threshold of 10.7 mm.

**Table 4:** Sensitivity, specificity, and AUC of the indices

parameters	Threshold	Sensitivity (%)	Specificity (%)	AUC	*p* -Value ^1^
Right					
Gonial angle	121.2 (120.3–121.3)	54.2 (54.2–54.2)	46.5 (43–48.8)	45.6	0.39
Condylar height	53.6 (53.1–54.7)	70.8 (68.8–75)	66.3 (64–73.3)	76.0	<0.01 b
Coronoid height	53 (52.9–53.2)	70.8 (70.8–72.9)	68.6 (66.3–69.8)	77.0	<0.01 b
Max ramus width	33.4 (33–33.5)	54.2 (52.1–58.3)	52.3 (48.8–53.5)	56.3	0.22
Min ramus width	24.3 (23.8–24.8)	52.1 (47.9–56.3)	51.2 (48.8–57)	49.8	0.97
Antegonial depth	1.6 (1.5–1.7)	60.4 (54.2–62.5)	57 (53.5–60.5)	62.8	0.014 b
Antegonial angle	166.6 (165.3–167.2)	45.8 (41.7–50)	51.2 (40.7–51.2)	42.2	0.138
**Left**	**Threshold**	**Sensitivity (%)**	**Specificity (%)**	**AUC**	***p*** **-Value**
Gonial angle	121.8 (121.2–122.2)	45.8 (43.8–52.1)	45.4 (43–46.5)	39.9	0.054
Condylar height	53.9 (53.6–54.2)	68.8 (64.6–72.9)	70.9 (68.6–72.1)	75.7	<0.01 b
Coronoid height	52.8 (52.6–53.3)	66.7 (69.8–72.9)	65.1 (61.6–64.6)	76.1	<0.01 b
Max ramus width	33.3 (33.2–33.6)	52.1 (47.9–58.3)	50 (48.8–51.2)	53.9	0.45
Min ramus width	24.6 (24.5–24.9)	47.9 (45.8–47.9)	46.5 (43–48.8)	47.2	0.58
Antegonial depth	1.5 (1.4–1.6)	58.3 (58.3–58.3)	57 (53.5–60.5)	63.5	0.01 b
Antegonial angle	167.3 (165.9–168.5)	41.7 (39.6–43.8)	41.9 (34.9–46.5)	38.2	0.024 b
**Total**	**Threshold**	**Sensitivity (%)**	**Specificity (%)**	**AUC**	***p*** **-Value**
Mean superior border of mental foramen	13.9 (13.8–14.1)	75 (72.9–77.1)	74.1 (70.6–80)	81.4	<0.01 b
Mean interior border of mental foramen	10.7 (10.6–11)	77.1 (70.8–81.3)	76.5 (72.9–81.2)	82.9	<0.01 b
Mean bicondylar width	150.9 (150.3–154.2)	68.8 (60.4–70.8)	54.1 (51.8–69.4)	62.5	0.01 b
Mean bigonial width	152.9 (151.8–155.5)	64.6 (58.3–66.7)	67.1 (60–72.9)	69.0	<0.01 b
Gonial angle	122.9 (121.9–124.2)	43.8 (41.7–54.2)	49.4 (45.9–60)	42.5	0.15
Condylar height	53.5 (53–54.8)	68.8 (68.8–70.8)	68.2 (62.4–76.5)	76.5	<0.01 b
Coronoid height	52.3 (51.9–53.4)	79.2 (70.8–81.3)	62.4 (57.6–74.1)	77.7	<0.01 b
Max ramus width	33.5 (33.2–33.8)	54.2 (50–60.4)	51.8 (48.2–52.9)	55.0	0.33
Min ramus width	24.6 (24–25.2)	47.9 (43.8–54.2)	45.9 (41.2–57.6)	48.4	0.76
Antegonial depth	1.6 (1.4–1.7)	60.4 (58.3–64.6)	58.8 (52.9–60)	65.4	0.003 b
Antegonial angle	166.1 (164.8–167.7)	43.8 (37.5–45.8)	42.4 (35.3–49.4)	38.1	0.023 b

Abbreviation: AUC, area under the curve.

a Analyzed with the help of binary logistic regression.

b AUC considered significant.

Indices such as gonial angle, maximum ramus width, and minimum ramus width had lower AUC values and were not statistically significant predictors of gender ( *p* > 0.05). These findings suggest that coronoid height and the inferior border of the mental foramen are the most reliable indicators for gender determination. The results from Tables 1 and 2 demonstrate that males exhibit significantly greater condylar height, coronoid height, bigonial width, and antegonial depth, while females have significantly greater gonial and antegonial angles. Table 3 indicates that logistic regression models can predict gender with high accuracy using condylar height, coronoid height, and mental foramen measurements. Table 4 confirms that coronoid height and the inferior border of the mental foramen provide the best discriminatory power for male gender identification, with high sensitivity and specificity. Overall, these findings highlight the effectiveness of certain mandibular indices in gender determination. With a gender distribution of 64% female and 36% male, indicating a higher proportion of females, the study analyzed mandibular morphometric variations for sex determination.

## Discussion

Identification of people and calculating demographic traits like age, sex, and ancestry can be performed using skeletal remains. Forensic determination capitalizes on the unique characteristics of bone. This study evaluates gender determination by employing mandibular indices. Eleven mandibular parameters were cross-validated, out of which four, namely, the bigonial width, coronoid height, antegonial angle, and the lower border of the mandible, showed the best precision for gender determination in the Pakistani cohort.

Gonial angle, maximum ramus width, and minimum ramus width were identified as the least predictive parameters for gender determination in the present study. In contrast to these findings, Esfehani et al 8 reported minimum ramus breadth as the most accurate parameter for gender determination in an Iranian population. Furthermore, Esfehani et al 8 recognized condylar height as a robust predictor of gender, a finding that aligns with the observations of Behl et al, 14 who also identified condylar height as a strong discriminatory parameter in a North Indian population. 8 14 These variations in predictive accuracy across different populations suggest that skeletal morphometric indices may exhibit population-specific differences, warranting further investigation in diverse demographic groups.

When comparing the findings on gonial angle across different studies, Esfehani et al 8 reported higher gonial angle values in the Iranian population. This variation suggests potential population-specific differences in mandibular morphology, highlighting the influence of genetic and environmental factors on craniofacial structures. 24 Our findings generally align with previous observations indicating a higher gonial angle in females. However, the accuracy of this parameter in gender differentiation was notably lower in our study compared with other investigations. When compared with studies conducted in Iran, our results revealed that both the threshold and mean values of the gonial angle were considerably higher. These differences suggest that the gonial angle may have a lower predictive value for gender determination in our studied population, underscoring potential population-specific variations in mandibular morphology. 8

Ojha et al collectively identified condylar height as one of the primary determinants in gender differentiation. Their findings reinforce the significance of condylar height as a key morphometric parameter in forensic and anthropological assessments of gender. 25 Similar to condylar height, coronoid height was identified as a distinct marker for gender differentiation in the Pakistani population. In the present study, coronoid height emerged as the third strongest indicator for gender determination, with a threshold of 53.7 mm and an accuracy of 77.7%. These findings align with those of Mehta et al, who reported a higher accuracy of 82.65% for coronoid height in an Indian population, suggesting potential regional variations in mandibular morphometric traits. 18

A study conducted on the Bagalkot population also identified coronoid height as a strong indicator for gender determination. However, the study's findings were based on a relatively smaller sample size of only 80 individuals, which may limit the generalizability of the results. In the present study, maximum and minimum ramus widths were among the parameters that did not demonstrate statistical significance in gender differentiation. Similarly, Ojha et al 25 and Ingaleshwar et al also assessed minimum ramus breadth as the least significant parameter in their respective studies, further supporting its limited predictive value across different populations. 25 Antegonial depth and the distance from the inferior border of the mental foramen emerged as two of the most reliable indices for gender determination in the Pakistani population. Among these, the distance from the inferior border of the mental foramen was identified as the most significant parameter, demonstrating an accuracy of 82.9% in gender classification. Furthermore, findings from Behl et al, 14 Chalazoniti et al, 26 and Esfehani et al 8 consistently indicate that bigonial width is greater in males than in females. The present study supports this observation, suggesting that the assumption of males exhibiting a larger bigonial width may hold true across diverse populations. 8 26

In contrast to bigonial width, bicondylar width was found to be greater in females than in males within the Pakistani population. The threshold value for bicondylar width was determined to be 150.9 mm, with values exceeding this threshold indicating male gender with an accuracy of 62.5%. This finding is inverse to observations reported in the Iranian population, suggesting potential regional variations in craniofacial morphology that may influence the predictive accuracy of bicondylar width in gender determination. 8 Studies conducted on other populations have reported higher bicondylar width values as indicative of male gender. The discrepancies observed in the literature compared with this study suggest that geographical variations and ethnic backgrounds play a significant role in forensic identification. Certain populations exhibit distinct skeletal patterns and morphometric measurements, which can serve as valuable markers in forensic analysis. This is particularly relevant in regions such as Pakistan, where antemortem data are often unavailable, emphasizing the importance of population-specific skeletal indices in forensic investigations. 4

Limitations of this study include its single-location nature, as it was conducted in Karachi, Pakistan, which restricts its generalizability to the broader Pakistani population. The findings may not fully represent mandibular morphometric variations across different regions and ethnic groups within the country. Additionally, the exclusion of edentulous patients may introduce potential biases, as age-related bone resorption and anatomical changes in the mandible were not accounted. This limitation may affect the applicability of the results to forensic cases involving older individuals or those with significant dental loss. The practical implications of these findings in forensic odontology are significant, particularly in regions where antemortem records are scarce.

The identified threshold values for mandibular indices, such as the inferior border of the mental foramen (82.9% accuracy) and coronoid height (77.7% accuracy), can be utilized in forensic casework to assist in gender identification of unknown skeletal remains. These values provide forensic experts with quantifiable benchmarks that can be used in both medicolegal investigations and mass disaster victim identification. For instance, in forensic cases where fragmented mandibles are recovered, the measured values can be compared against these thresholds to establish the likelihood of male or female identity, thereby narrowing down the pool of potential matches in missing persons investigations. Additionally, the findings may aid forensic anthropologists in creating population-specific forensic databases, improving the accuracy of sex estimation models in South Asian populations.

Future research should aim to refine these predictive models by incorporating larger and more diverse population samples across different regions of Pakistan to improve the generalizability of findings. Additionally, examining other mandibular parameters, such as symphyseal height and ramus flexure, could enhance predictive accuracy. Another promising direction is the integration of machine learning algorithms to develop automated forensic identification systems based on panoramic radiographs. Utilizing artificial intelligence in forensic odontology may allow for greater precision, efficiency, and reliability in gender determination, particularly in cases where skeletal remains are incomplete or fragmented. Furthermore, future studies should explore age-related changes in mandibular morphology to assess the applicability of these indices across different age groups, ensuring the robustness of forensic gender determination methods.

## Conclusion

Panoramic radiographs and morphometric parameters can be used in forensic odontology for accurate and efficient gender determination in the specific socio-cultural context of Pakistani population.

## References

[1] Lashin H I Sharif A F Ghaly M S El-Desouky S S Elhawary A E Bridging gaps in age estimation: a cross-sectional comparative study of skeletal maturation using Fishman method and dental development using Nolla method among Egyptians Int J Legal Med 2025 139 02 695 714 39760867 10.1007/s00414-024-03394-x PMC11850478

[2] Lashin H I Eldeeb B S Ghonem M M Sex identification from foramen magnum using computed tomography scanning in a sample of Egyptian population J Forensic Radiol Imaging 2019 19 100341

[3] Forrest A Forensic odontology in DVI: current practice and recent advances Forensic Sci Res 2019 4 04 316 330 32002490 10.1080/20961790.2019.1678710 PMC6968523

[4] Baqai H S Zaidi S JA Baig Q A Bashir M B Anwar M Ansari A S Maintenance of dental records and awareness of forensic odontology among pakistani dentists: a mixed-method study with implications for dental data repository BMC Oral Health 2023 23 01 783 37875855 10.1186/s12903-023-03500-2 PMC10594786

[5] Arthanari A Senthilkumar A Ramalingam K Prathap L Ravindran V Exploring age and gender identification through mandibular parameters using orthopantomography: an observational study Cureus 2024 16 03 e55788 38590503 10.7759/cureus.55788 PMC11000035

[6] Chovalopoulou M E Valakos E Nikita E Skeletal sex estimation methods based on the Athens collection Forensic Sci 2022 2 04 715 724

[7] Kim H J The mandible: an atlas of osteological and radiological anatomy Anat Cell Biol 2022 55 01 1 2 35354671 10.5115/acb.22.0316 PMC8968226

[8] Esfehani M Ghasemi M Katiraee A Forensic gender determination by using mandibular morphometric indices an Iranian population: a panoramic radiographic cross-sectional study J Imaging 2023 9 02 40 36826959 10.3390/jimaging9020040 PMC9960296

[9] Hatipoğlu F P Arıcıoğlu B Hatipoğlu Ö Köse T E Günaçar D N Prediction of root canal lengths and pulp volume of the maxillary permanent first molar based on stature, crown diameters, and facial morphometry Anat Sci Int 2023 98 03 454 462 37079264 10.1007/s12565-023-00727-5

[10] Pertek H Kamaşak M Kotan S Hatipoğlu F P Hatipoğlu Ö Köse T E Comparison of mandibular morphometric parameters in digital panoramic radiography in gender determination using machine learning Oral Radiol 2024 40 03 415 423 38625432 10.1007/s11282-024-00751-9

[11] Saini V Chowdhry A Mehta M Sexual dimorphism and population variation in mandibular variables: a study on a contemporary Indian population Anthropol Sci 2022 130 01 59 70

[12] Karmarkar P H Mhapuskar A Prasad Hiremutt D R Kale I P Tepan M Rao P Mandibular ramus: an indicator for gender determination Cureus 2023 15 01 e34192 36843791 10.7759/cureus.34192 PMC9957585

[13] Hemgude P Sabane A Shinde S Mandibular ramus: an indicator for sex determination-a digital radiographic study Neuro Quantology 2022 20 09 4428 4436

[14] Behl A Grewal S Bajaj K Baweja P Kaur G Kataria P Mandibular ramus and gonial angle - identification tool in age estimation and sex determination: A digital panoramic radiographic study in north indian population J Indian Acad Oral Med Radiol 2020 32 01 31 36

[15] More C B Vijayvargiya R Saha N Morphometric analysis of mandibular ramus for sex determination on digital orthopantomogram J Forensic Dent Sci 2017 9 01 1 5 28584466 10.4103/jfo.jfds_25_15 PMC5450475

[16] Garg R Jacky T Yung TGH Li YW Brahanudin TMBT To assess the usefulness of the mandibular ramus in determining age and gender among Malaysians in digital OPGs J Int Dent Med Res 2021 14 04 1472 1477

[17] Verma P Mahajan P Puri A Kaur S Mehta S Gender determination by morphometric analysis of mandibular ramus in sriganganagar population: a digital panoramic study Indian J Dent Res 2020 31 03 444 448 32769281 10.4103/ijdr.IJDR_547_17

[18] Mehta H Bhuvaneshwari S Singh M Nahar P Mehta K Sharma T Gender determination using mandibular ramus and gonial angle on OPG J Indian Acad Oral Med Radiol 2020 32 02 154 158

[19] Khan M I Gopal T Laxmikanth S M Varughese M A Radiographic investigation of the antegonial angle, antegonial depth, gonial angle, and the ramus height to estimate the gender and growth patterns in orthodontic patients J Indian Acad Oral Med Radiol 2024 36 02 169 172

[20] Ingaleshwar P Bhosale S Nimbulkar G Britto F Chandrappa P R Hosur M B Mandibular ramus- an indicator for gender determination: a digital panoramic study in Bagalkot population J Oral Maxillofac Pathol 2023 27 01 66 70 37234315 10.4103/jomfp.jomfp_62_22 PMC10207222

[21] Kathoju M Guttikonda V Age estimation using mandibular ramus and gonial angle using digital orthopantamogram Int J Forensic Odontol 6 01 27 31

[22] Shah P H Venkatesh R More C B Vaishnavee V Age- and sex-related mandibular dimensional changes: a radiomorphometric analysis on panoramic radiographs Indian J Dent Res 2020 31 01 113 117 32246692 10.4103/ijdr.IJDR_327_18

[23] Magar S S Magar S P Rangari P Mishra B P Chhabada A K Age estimation by antegonial angle, depth measurement and tooth coronal index by digital panoramic radiography Int J Health Sci (Qassim) 2022 6(S3):998 1005

[24] Nimigean V Gherghiţă O R Păun D L Morphometric study for the localization of the mental foramen in relation to the vertical reference plane Rom J Morphol Embryol 2022 63 01 161 168 36074680 10.47162/RJME.63.1.17 PMC9593127

[25] Ojha B Bajracharya D Koju S Maharja N Saha A Baral R Mandibular parameters as a predictor of sex: a digital orthopantomogram study J Kathmandu Med Coll 2021 10 04 218 223

[26] Chalazoniti A Lattanzi W Halazonetis D J Shape variation and sex differences of the adult human mandible evaluated by geometric morphometrics Sci Rep 2024 14 01 8546 38609399 10.1038/s41598-024-57617-7 PMC11014969

